# A Ferrofluid with Surface Modified Nanoparticles for Magnetic Hyperthermia and High ROS Production

**DOI:** 10.3390/molecules27020544

**Published:** 2022-01-15

**Authors:** Oscar Cervantes, Zaira del Rocio Lopez, Norberto Casillas, Peter Knauth, Nayeli Checa, Francisco Apolinar Cholico, Rodolfo Hernandez-Gutiérrez, Luis Hector Quintero, Jose Avila Paz, Mario Eduardo Cano

**Affiliations:** 1 Centro Universitario de Ciencias Exactas e Ingenierías, Universidad de Guadalajara, Marcelino García Barragán 1421, Col. Olímpica, Guadalajara C.P. 44430, Jalisco, Mexico; ocervantesa@gmail.com (O.C.); ncasa@hotmail.com (N.C.); 2 Centro Universitario de la Ciénega, Universidad de Guadalajara, Av. Universidad 1115, Col. Linda Vista, Ocotlan C.P. 47810, Jalisco, Mexico; zlopez@gmx.net (Z.d.R.L.); cbl08@gmx.de (P.K.); sarai.melendez@alumnos.udg.mx (N.C.); pako.cholyko@gmail.com (F.A.C.); jocmos@cuci.udg.mx (J.A.P.); 3 Centro de Investigación y Asistencia en Tecnología y Diseño del Estado de Jalisco, A.C. Av. Normalistas 800 Colinas de La Normal, Guadalajara C.P. 44270, Jalisco, Mexico; rhgutierrez@ciatej.mx; 4 Centro Universitario de Ciencias Económico Administrativas, Universidad de Guadalajara, Periférico Norte 799, Col. Los Belenes, Zapopan C.P. 45100, Jalisco, Mexico; hectorquintero@yahoo.com

**Keywords:** superparamagnetism, hyperthermia, ferrofluid, colloidal-stability, ROS

## Abstract

A ferrofluid with 1,2-Benzenediol-coated iron oxide nanoparticles was synthesized and physicochemically analyzed. This colloidal system was prepared following the typical co-precipitation method, and superparamagnetic nanoparticles of 13.5 nm average diameter, 34 emu/g of magnetic saturation, and 285 K of blocking temperature were obtained. Additionally, the zeta potential showed a suitable colloidal stability for cancer therapy assays and the magneto-calorimetric trails determined a high power absorption density. In addition, the oxidative capability of the ferrofluid was corroborated by performing the Fenton reaction with methylene blue (MB) dissolved in water, where the ferrofluid was suitable for producing reactive oxygen species (ROS), and surprisingly a strong degradation of MB was also observed when it was combined with H_2_O_2_. The intracellular ROS production was qualitatively corroborated using the HT-29 human cell line, by detecting the fluorescent rise induced in 2,7-dichlorofluorescein diacetate. In other experiments, cell metabolic activity was measured, and no toxicity was observed, even with concentrations of up to 4 mg/mL of magnetic nanoparticles (MNPs). When the cells were treated with magnetic hyperthermia, 80% of cells were dead at 43 °C using 3 mg/mL of MNPs and applying a magnetic field of 530 kHz with 20 kA/m amplitude.

## 1. Introduction

The stable surface binding between iron oxide nanoparticles and catechol (or 1,2-benzenediol) derived compounds is well documented [1]. In colloidal suspensions, their long-time stability has been studied [2], as well as their good molecular anchoring properties [1,2,3]. The catechol derivative 3,4-dihydroxyphenylalanine (DOPA) has been found in mussel adhesive proteins, proteins that mussels use to adhere to nearly any surface, which inspired the use of catechol and other derivatives in biocompatible and stable nanoparticles [4]. In the biomedical field, colloidal systems of superficially modified magnetic nanoparticles (MNPs) for anchoring drugs are attractive alternatives for cancer diagnostics and therapies [5]. In polymer science, some compounds derived from catechol are an interesting platform for developing biometric functional materials [6], novel adhesive materials [7], contaminating compound removers [8], or antioxidant products [9].

The production of reactive oxygen species (ROS) is directly related to the oxidative capability of the compounds internalized by living organisms. Controlled induction of ROS by new substances such as MNPs or ferrofluids can be an attractive choice for cancer treatment. In particular, the use of thermo-therapies complemented with nanomaterials capable of destroying tissues by heating (necrosis) and ROS (apoptosis) has opened a new field of opportunities in the area of nanomedicine [10]. In contrast, the excessive production of ROS represents a serious risk factor for producing other diseases such as cancer [11] or Parkinson’s disease [12]. When this overproduction of ROS occurs in a living system, the internalization of antioxidant substances is the ideal mechanism to reach the normal production rate [13].

In the field of magnetic hyperthermia (MHT) for cancer therapy, dopamine is one of the catechol-derived coatings that has been frequently used [14,15,16]. Recently, an easily prepared ferrofluid using dopamine coated MNPs has been reported [17], which has not only very low cytotoxicity but also a high power absorption density. In that work, good cancer therapy results are obtained in vivo experiments. Another study demonstrated that, at room temperature, ROS production is regulated when MNPs are coated with different organic compounds (including dopamine) when compared with uncoated MNPs [18]. These results suggest that the ROS production using iron oxide MNPs can be modulated through their surface modification.

In MHT, the absorbed power density *P* from an alternant magnetic field, which is transmitted to a volume V using a ferrofluid, can be modeled through the linear response theory Equation (1) [19,20]. This approximation involves a set of ideal physical conditions because it takes into account perfectly suspended MNPs (colloidal suspension) identical in diameter σ or volume (*Vp*), plus other magnetic properties of superparamagnetic monodomains. The explicit parameters involved are the complex magnetic susceptibility of the ferrofluid $\chi_{F}^{’’}$, the frequency *f* of the irradiated magnetic field, its quadratic amplitude *H*^2^, and the magnetic permeability of the free space *µ*_0_ (given by Equation (2)):

(1) $$P=\mu_{0}\pi \chi_{F}^{’’}fH^{2}$$

(2) $$\mu_{0}=4\pi \;\times 10^{-7}N/A^{2}$$

From a more practical point of view, the parameter *P* can be determined by performing calorimetric trials employing magnetic induction heating on phantoms of water. For this purpose, very diluted ferrofluids are prepared with fractions η less than 1% of MNPs suspended; thus, the effective density and specific heat are approximated to *ρ_F_* = 1.0 g/cm^3^ and to *c_v_* = 4.186 J/(g*K), respectively. Furthermore, the procedure consists of the previous estimation of the specific absorption rate *SAR* (given by Equation (3)) [21] with fixed parameters *f* and *H*. Later, the *SAR* is transformed in *P* through the Equation (4) [22]:

(3) $$SAR=\frac{c_{v}}{m_{np}}\frac{dT}{dt}\;$$

(4) $$P=\rho_{F}\times SAR\;$$

The production of ROS by iron oxide nanostructures is widely characterized through the classical (homogeneous) Fenton reaction. When hydrogen peroxide is reduced by ferrous iron species in a single electron transfer, then hydroxyl radicals are produced with sufficient energy to oxidize organic compounds, and this Fe^3+^/Fe^2+^ redox cycle is expressed in the following reactions:

Fe^3+^ + H_2_O_2_ → Fe^2+^ + HO_2_^•^ + H^+^

Fe^2+^ + H_2_O_2_ → Fe^3+^ + OH^−^ + ^•^OH

On the other hand, a general explanation concerning the mechanism of the heterogeneous Fenton reaction represents a difficult task because it depends on the selected catalyst or degrading materials. For example, the degradation of methylene blue (MB) was recently modeled and evaluated using MNPs of magnetite coated with a catechol-derived compound, and a new heterogeneous reaction mechanism is widely explained in [23]. In that work, it was shown that the negative charge of the coating induces the adsorption of MB and the catalytic activation of H_2_O_2_. Then, the MB is easily oxidized by the ·OH radicals generated on the surface of the magnetite, which contributes to its degradation.

To our knowledge, few works delve into the changes induced by pure catechol (a strong antioxidant) to the physical and chemical properties of ferrofluids with iron oxide MNPs. Thus, the main aim of this work was the synthesis and experimental analysis of a colloidal suspension of catechol-coated MNPs, highlighting the physicochemical changes due to the modification of their surface. Its suitability was also analyzed for magneto-thermal therapies by means of the typical physicochemical characterization, but experiments are also currently performed to determine cytotoxicity, oxidation capability, and intracellular ROS production.

## 2. Results and Discussion

**Figure 1:**
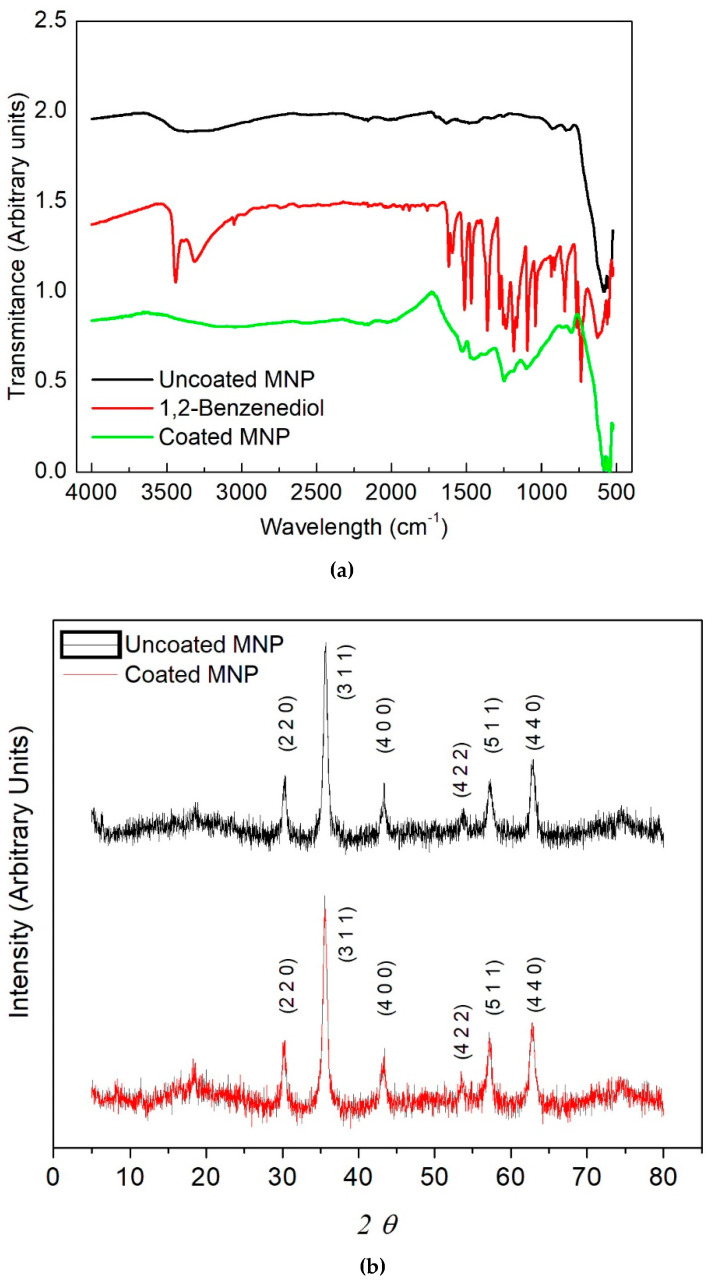
(**a**) FTIR spectra of uncoated MNPs, catechol and coated MNPs; (**b**) XRD spectra of both coated and uncoated MNPs.

The FTIR spectra of uncoated and coated MNPs are presented in Figure 1a. In relation with the uncoated MNPs, the obtained spectrum exhibits two main minimum values at ν = 3363.30 cm^−1^ (between 2656 and 3645 cm^−1^) and 589.46 cm^−1^, which are associated with the stretching of the –OH group and the Fe–O bonding, respectively. Likewise, the spectrum of catechol exhibits strong wavelengths at ν = 3442.40 cm^−1^ and 3315.09 cm^−1^ related with intermolecular bindings; the peaks at 1618.14, 1593.66, 1512.53 and 1467.67 cm^−1^ indicate motions of the C=C ring; the peak at 1360.37 cm^−1^ is related with a motion of the –C–O–H plane and the signals at 1278.13, 1254.07 and 1237.15 cm^−1^ describe motion in the –CH plane; the peaks at 1183.89 and 1163.81 cm^−1^ are associated with motions in the –C–O–H plane; the spikes at 1093.34 and 1039.55 cm^−1^ are related with motions of the –CH plane; the signals at 936.23, 915.66, and 847.48 cm^−1^ indicate motions outside of the C=C plane. Regarding the spectrum of the coated MNPs, it exhibits a clear widening between 3640 cm^−1^ up to 2257 cm^−1^ having a minimum in 3150 cm^−1^ shifted to the left with respect to the last two FTIR spectra. These characteristics are related to the interactions between the deprotonated oxygen molecules of catechol with the Fe^2+^ and Fe^3+^ ions, which are placed in the octahedral positions of the magnetite. While the width of the signal in 1526.24, 1447.82, 1248.48, 1099.86, 801.38, and 585.38 cm^−1^ is conserved, very weak intensities are observed at 1380 and 1186 cm^−1^, which indicates the binding of catechol to the surface of the MNPs [1,2].

In the same manner, the X-ray diffraction spectra of coated and uncoated MNPs are depicted in Figure 1b. As can be observed, the sample with uncoated MNPs presents peaks at 30.27°, 35.66°, 43.33°, 53.75°, 57.27°, and 62.8°, which are associated with the Miller indices (2 2 0), (3 1 1), (4 0 0), (4 2 2), (5 1 1), and (4 4 0), respectively. According to JCPDS 19-0629, this observation corresponds to magnetite (spatial group Fd3m, # 227). Now, by analyzing the spectrum of the sample of the coated MNPs, the same peaks and indices were registered. Indeed, no shifting and widening were observed in the different phases of magnetite; this indicates the conservation of its inverse spinel structure despite the coating process. The Scherrer–Debye equation (Equation (5)) was applied to the width of the signals (at half height *β*) in the Bragg angle *θ* corresponding to the plane (3 1 1). Hence, *τ* = 13.0 nm of the crystal size is found for uncoated and coated samples, previously assuming the form factor *κ* = 0.94 and wavelength *λ* = 0.154 nm.

(5) $$\tau =\frac{\kappa \lambda }{\beta \cos\theta }$$

**Figure 2:**
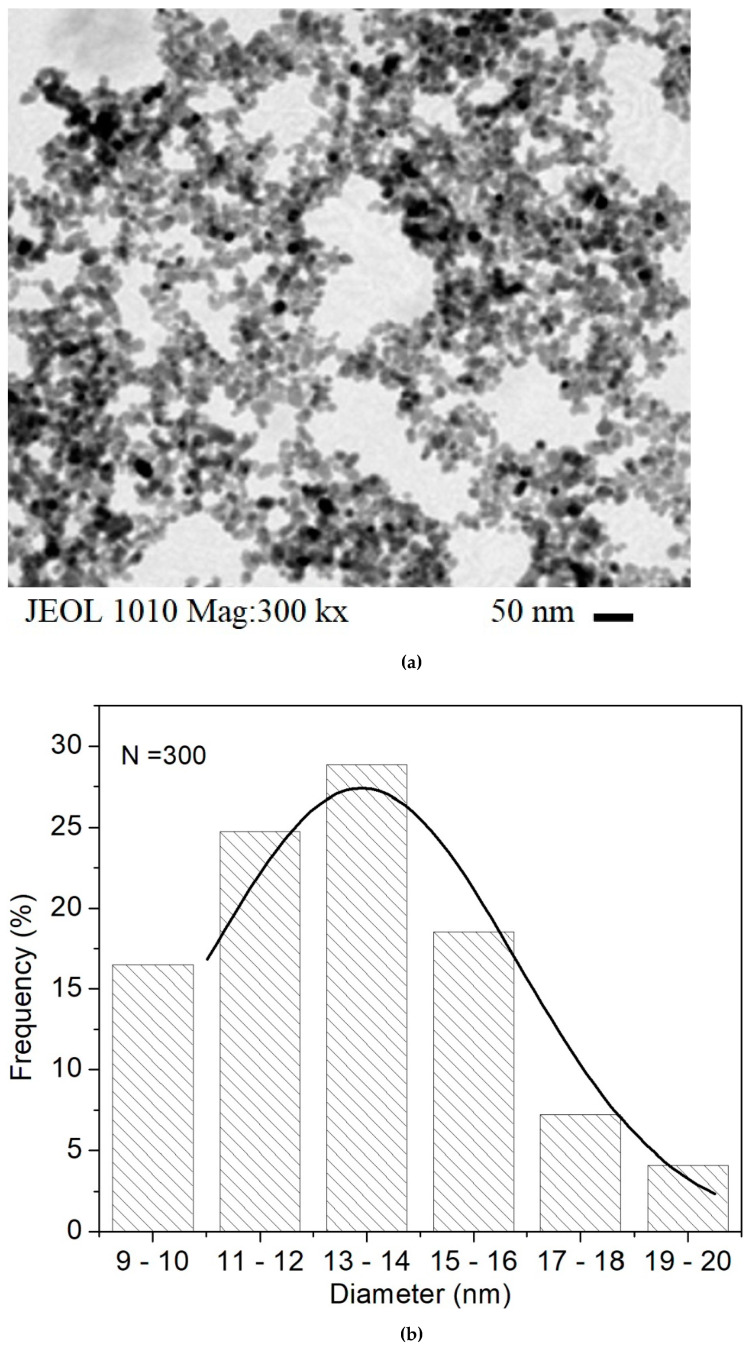
(**a**) A typical TEM micrograph (300 kx) of coated MNPs and (**b**) the corresponding bars plot and Gaussian fit.

On the other hand, a typical TEM micrograph of the coated MNPs samples is shown in Figure 2a. As can be observed, the minimal scale utilized is 50 nm (with 300 kx of magnification) and spherical-like gray and dark dots appear in the image. After an analysis of a set of micrographs employing the ImageJ software (https://imagej.nih.gov/ij/, accessed on 1 June 2021), a bar plot was made to determine the particle diameter σ. In Figure 2b, the histogram is shown using *n* = 300 MNPs, in which the central value of each bar is fitted to a Gaussian distribution function, reaching σ = 13.5 ± 5 nm. This diameter size is slightly larger than the crystal size measured by XRD, also some polydispersity of the particle sizes is observed, but this non-uniformity is the expected behavior due to the synthesis method of co-precipitation [16,17,24].

**Figure 3:**
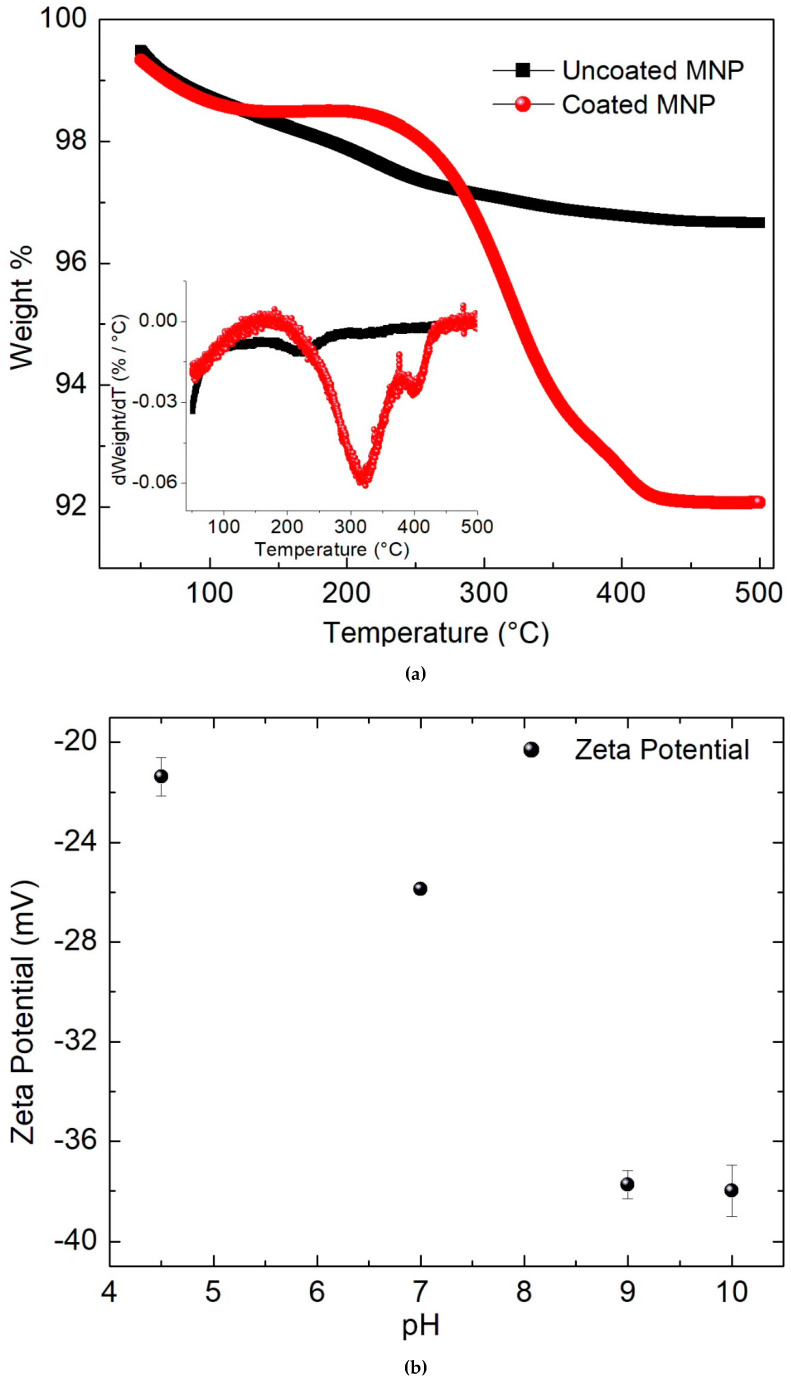
(**a**) Dependence of temperature on the mass loss of the uncoated and coated MNPs, the inset are the derivatives of the mass loss plot, and (**b**) the zeta potential variation between pH = 4.5 and 10.0.

Concerning TGA measurements, Figure 3a displays the mass loss of uncoated and coated MNPs while the temperature is continuously increased. As can be observed, the uncoated MNPs undergo a 3.25% mass loss associated with the water linked to the structure of the iron oxide. In contrast, the thermogram of the coated MNPs demonstrates a 7% mass loss, corresponding to the catechol bonded to the surface of the magnetite. In this inset of Figure 3a, the derivatives of the plots of Figure 3a are displayed. Then, the observed thermal transitions in uncoated MNPs are shown at 210 °C, due to the strong binding of water with the iron oxide, while, in coated MNPs, this transition is shown at 173 °C. In addition, in coated MNPs, other transitions at 310 °C and 380 °C are due to the strong catechol binding with the iron oxide. As no other organic materials are present in this ferrofluid (which was washed four times), it is reasonable to suppose that boiled catechol had completely adhered to the MNPs. Thus, the volume ratio of coated and uncoated samples is given by Equation (6):

(6) $$\frac{V_{coated}}{V_{uncoated}}=\left(\frac{0.93\rho_{catechol}}{a\rho_{catechol}+b\rho_{catechol}}\right)$$

where *a* = 0.93 and *b* = 0.07 are the mass fraction of magnetite and catechol and also *ρ_catechol_* = 1.34 g/cm^3^ and *ρ_magnetite_* = 5.18 g/cm^3^, are their respective mass density. Assuming that the MNPs have spherical shapes, the thic1kness of the catechol shell with the formula (Equation (7)):

(7) $$\Delta \sigma =\left({\left(\frac{V_{coated}}{V_{uncoated}}\right)}^{-\frac{1}{3}}-1\right)\sigma_{uncoated}$$

is deduced, where only 1.2 nm of thickness is estimated.

The results about the magnetic properties and colloidal stability of the ferrofluids are now discussed. In Figure 3b, the zeta potential measurements for different pH are displayed; according to the measured amplitudes, it is remarkable that no clusters of coated MNPs were formed. The higher measurements are observed for pH > 7 reaching approximately −38 mV (9 < pH < 10); these measurements indicate good stability of the ferrofluid. During three months of observation, no sedimentation of the coated MNPs was able to be observed (at room temperature). In contrast, for pH = 7 and 4.5, the intensities are 26 mV and 21 mV, respectively, which involves a moderated (but adequate) colloidal stability [17]. Thus, the coated MNPs are dispersed in the liquid medium and after 2 h they are partially precipitated without clusters forming. Indeed, MNPs always can be easily resuspended with only one shaking motion of the container. In particular, this slow sedimentation reached at pH = 7 is the desired property for in vitro experiments of cytotoxicity because the physical contact between the MNPs and the cell membranes is increased; as a consequence, better endocytosis can be expected. Thus, the higher endocytosis promotes a more reliable measurement of toxicity and represents the starting point to induce apoptosis (or another non-necrotic pathway) in cells, in magnetic hyperthermia assays.

**Figure 4:**
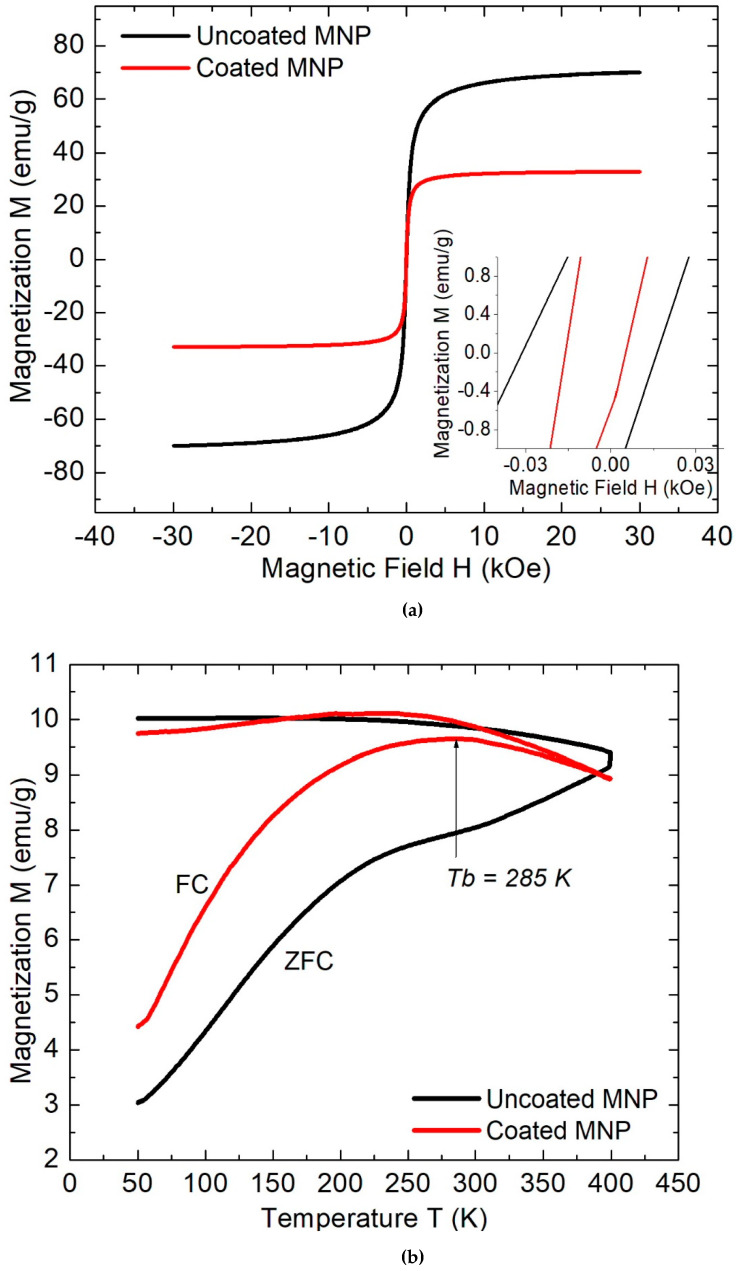
(**a**) Hysteresis loops of coated and uncoated MNPs and (**b**) their ZFC-FC plots.

Concerning the VSM measurements, which are obtained under room temperature conditions, the hysteresis loops of the samples of uncoated and coated MNPs are displayed in Figure 4a. The magnetic saturation observed for coated MNPs (*M_s_coated_* = 34 emu/g) is approximately 50% less than the uncoated MNPs (*M_s_uncoated_* = 70 emu/g), and this intensity is very similar to that obtained for other coated MNPs, in which some catechol derived compounds have also been used [16,17].

Additionally, by analyzing the hysteresis loop of Figure 4a, non-significant differences are measured in the remanence *M_r_coated_* = 1.7 emu/g and the reached coercive field (*H_c_coated_* = 10 Oe) is 50% diminished. The magnetic saturation is higher in uncoated MNPs due to the absence of diamagnetic material, which conversely is covering the surface of the coated MNPs. Moreover, the observed low coercivity and remanence in both samples (see insets of the same plot) indicate the possibility of a superparamagnetic behavior. This magnetic ordering can be expected due to the small diameters determined with TEM and XRD, which are less than 35 nm [25,26]. To extend the magnetic analysis, the ZFC-FC measurements are displayed in Figure 4b. As is observed in the ZFC trace of uncoated samples, an inflection point is observed at 225 K, but the thermal fluctuations are not sufficient to reach the Néel relaxation effect, and no deflections are present. This absence of a blocking temperature indicates a magnetic blocked state of the magnetization. Nevertheless, a blocking temperature of Tb = 285 K with reversibility temperature Tr = 390 K is reached in coated MNPs, demonstrating a predominant superparamagnetic behavior (which could be silent in the uncoated MNPs samples). Furthermore, the slow increase of the ZFC magnetization trace with temperature indicates the presence of polydispersity of particle sizes. Indeed, this non-uniformity shows qualitatively good accordance with the bar plot distribution of Figure 2b, estimated from TEM measurements. Thus, the catechol cover regulates the superparamagnetic state of the MNPs, but due to the narrow thickness estimated (1.2 nm), this effect could not be completely associated with the diminution of dipolar inter-particle interactions. A possible explanation can be associated with a greater number of large grains in the uncoated samples [27], which also increases the magnetic saturation and coercivity in the hysteresis loop of Figure 4a, and, conversely, the catechol coating avoids that clusterization due to negative electrostatic repulsions of hydroxyl groups formed around the Fe_3_O_4_ [23]. Other possible physical explanations must be explored considering changes in the anisotropic energy of both presentations of MNPs (coated and uncoated) [15] and/or the possible existence of exchange interactions [28]. Indeed, several authors have observed the same magnetic modulating phenomenon with catechol-derived coatings [15,17,29,30].

**Figure 5:**
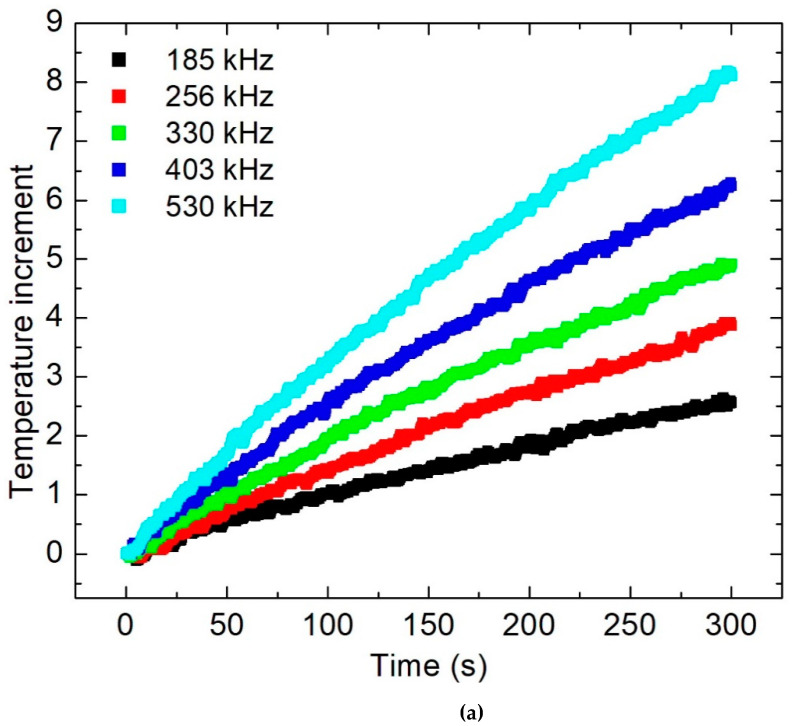

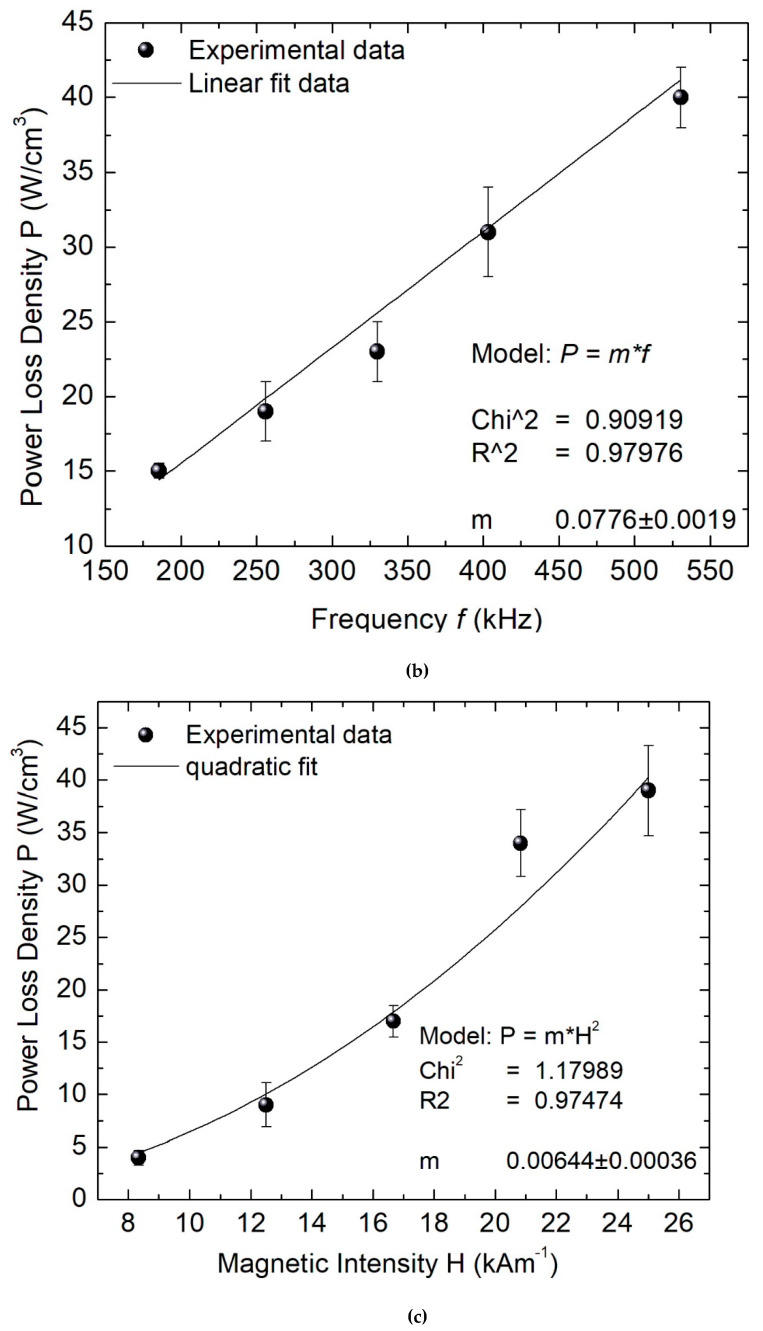
Dependences of: (**a**) *T* vs*. t* for several values *f*; (**b**) *P* vs. *f* including a linear fit to experimental data; and (**c**) *P* vs. *H* including a quadratic fit to experimental data.

Regarding the calorimetric procedure to analyze the ferrofluid, a fraction ɳ = 0.0034 of coated MNPs suspended in the liquid medium was previously measured. Additionally, to determine the background heating of all the samples, an Eppendorf tube sample of pure water with 1.0 g of mass was prepared for this use. Thus, a typical curve of the temperature increment for 5 min is presented in Figure 5a, where the background heating has been removed (0.001 °C/s approximately). In these experiments, *H* = 20 kA/m was the used magnetic intensity, while *f* increases from 185 kHz up to 530 kHz. As can be observed, the temperature slope and the maximum reached values increase along with *f*. Following the recommendations of [31], the temperature slope $\frac{dT}{dt}$ in each curve is determined over the time interval 20 s < *t* < 40 s. Then, the SAR is computed (for each *f*) using Equation (3) and this parameter is employed in the formula (Equation (4))to obtain the correlation of *P* vs. *f* as displayed in Figure 5b. In this plot, the bars are the standard deviation of each measurement and the solid line is a linear regression fit to the experimental data. Then, the parameter *P* increases at 0.078 ± 0.002 W/cm^3^ per kHz, and χ^2^ = 0.91 is the reached quality factor of the data fit. This statistical value indicates a good agreement of *P* with the theoretical linear response model of Rosensweig Equation (1); additionally, the reached slope is nearly 1.7 times higher that obtained using a ferrofluid of oleic acid-coated MNPs irradiated with identical magnetic fields [32]. Similar calorimetric experimental procedures and data processing are followed to determine the dependencies of *T* vs. *t* (data not shown) and then *P* vs. *H*. For this purpose, *f* = 329.8 kHz is a fixed parameter and *H* (in kA/m units) covers the interval 8.33 kA/m < *H* < 25 kA/m, with 4.17 kA/m increments. Therefore, the physical magnitudes *SAR* and *P* are computed; and this last is depicted in Figure 5c. The measurements include their standard deviations, and a quadratic regression is fitted to the experimental data using the formula (Equation (8)):

(8) $$P=m\times H^{2}$$

The obtained parameter *m* indicates the increase of *P* at the rate of 0.064 ± 0.004 W/cm^3^ per kA/m with χ^2^ = 1.18, indicating a moderate agreement with the quadratic dependence of *H*, which is also described in the Rosensweig theory. In these experiments, the maximum reached *P* and *SAR* are 40% less than the reported values for another catechol-derived ferrofluid, where higher intensities *H* were applied [33].

**Figure 6:**
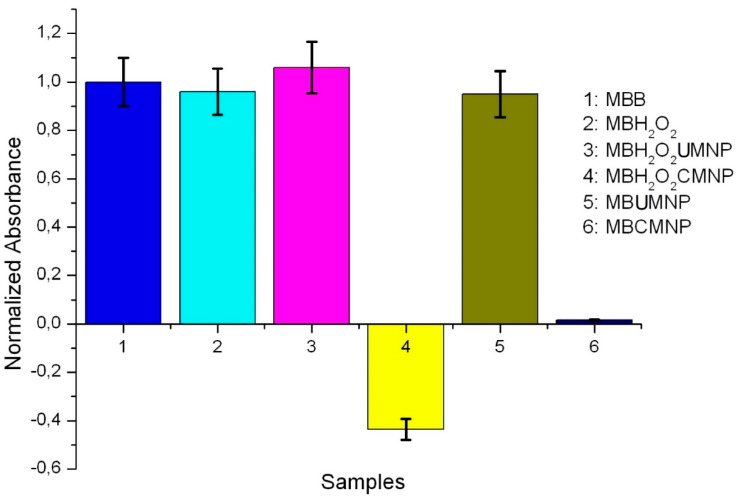
Bar plot representing the absorbance of the samples MBB, MB H_2_O_2_, MB H_2_O_2_ UMNP, MB H_2_O_2_ CMNP, MB UMNP, and MB CMNP.

The results of the Fenton reaction are displayed in the bar plot of Figure 6. According to this plot, the absorbance of samples MBB, MB H_2_O_2_, MB H_2_O_2_ UMNP, and MB UMNP do not exhibit significant differences, as if the Fenton reaction is slightly inhibited. The absorbance observed in MB UMNP is due to the absence of H_2_O_2_, which is necessary to carry out the Fenton reaction, and the absorbance of MB H_2_O_2_ UMNP is due to the non-optimal pH of the sample (pH ≈ 4), which limits the catalytic activity of the iron oxide [34]. In contrast, a very low absorbance is measured in MB CMNP, and, surprisingly, it is strongly diminished when H_2_O_2_ and catechol-coated MNPs are incorporated. The strong diminution in the absorbance of MB H_2_O_2_ CMNP exhibits high ROS production, which is due to the catalytic activity of catechol, generating ·OH by activation of H_2_O_2_ with consequent degradation of MB, showing good agreement with the degradation observed using the polycatechol coated MNPs reported in [23]. On the other hand, the low absorbance in MB CMNP may be due to the early degradation of MB adsorbed in CMNP through electrostatic adsorption [23]. Moreover, it is known that catechol can directly reduce Fe^3+^ to Fe^2+^ and it is transformed to quinone, which is an oxidant agent capable of degrading MB [35].

**Figure 7:**
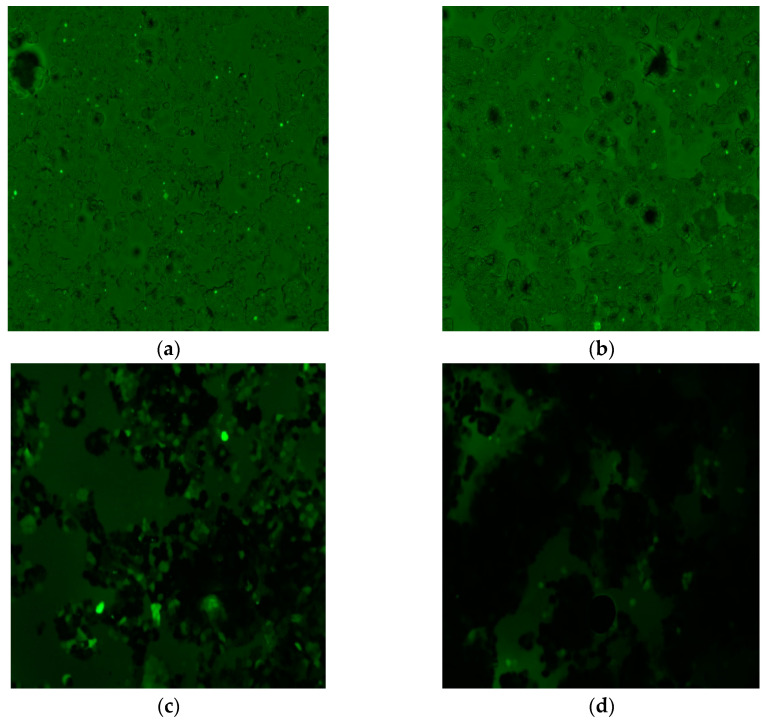
A sequence of microscope images of the HT-29 cells using a green light filter and a blue light source of the labeled samples: (**a**) NC; (**b**) PC; (**c**) DCF H_2_O_2_ UMNP; (**d**) DCF UMNP.

The production of ROS due to the UMNP is explained in the sequence of typical images of Figure 7a–d obtained through an epifluorescence microscope. The NC sample is shown in Figure 7a, where only sporadic emitting points of fluorescence in some cells are observed, corresponding to a threshold level of ROS production as a proper cell signaling. Additionally, although the PC sample contains H_2_O_2_, its fluorescence as registered in Figure 7b is very similar to that of NC. This is explained as the cells having an active defense mechanism to maintain a low concentration of ROS; for instance, the catalase enzyme protects the cells against H_2_O_2_. In contrast, when the UMNP are added, a higher number of fluorescent cells were registered than for the samples NC and PC. As can be observed in the image of sample DCF H_2_O_2_ UMNP displayed in Figure 7c, some cells appear to be completely green, few cells emitted intense green color and the majority of these have minor intensity; in this case, the UMNP is directly exposed to H_2_O_2_, then a Fenton reaction is carried out and ·OH are produced. Meanwhile, the number of fluorescent cells in the sample DCF UMNP is drastically diminished due to the MNPs are not being mixed with H_2_O_2_, as can be observed in the image of Figure 7d.

**Figure 8:**
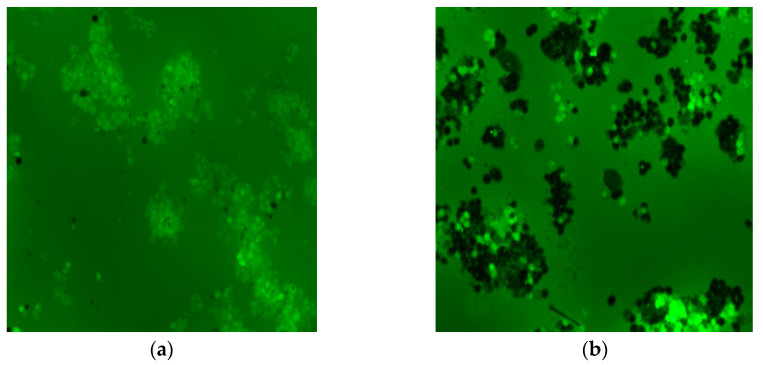
Microscope images of the HT-29 cells using a green light filter and the blue light source, of the labeled samples: (**a**) DCF H_2_O_2_ CMNP and (**b**) DCF CMNP.

In Figure 8a,b, the production of ROS due to the CMNP can be qualitatively explained, in both images, the cells exhibit normal shapes but significantly fewer confluences than that observed in Figure 7a–d. The image of the sample DCF H_2_O_2_ CMNP is shown in Figure 8a, where a nearly homogeneous fluorescence emission of all the cells is observed. This homogeneity is not present in the control samples (NC and PC), and also not in the cells of the sample DCF CMNP displayed in Figure 8b. Nevertheless, the sample DCF CMNP contains the brightest fluorescent cells. However, both samples exhibit a high rise of intracellular ROS induced by the catechol coating of the MNPs, which is increased when H_2_O_2_ is added. It is known that a Fenton reaction occurs in the cells when iron oxide MNPs are internalized [36]. During the degradation of the endocytosed CMNP, a partial separation of catechol from the magnetite can be induced, where the catechol can reduce Fe^+3^ to Fe^+2^, and it is modified to quinone, producing a high intracellular level of ROS.

**Figure 9:**
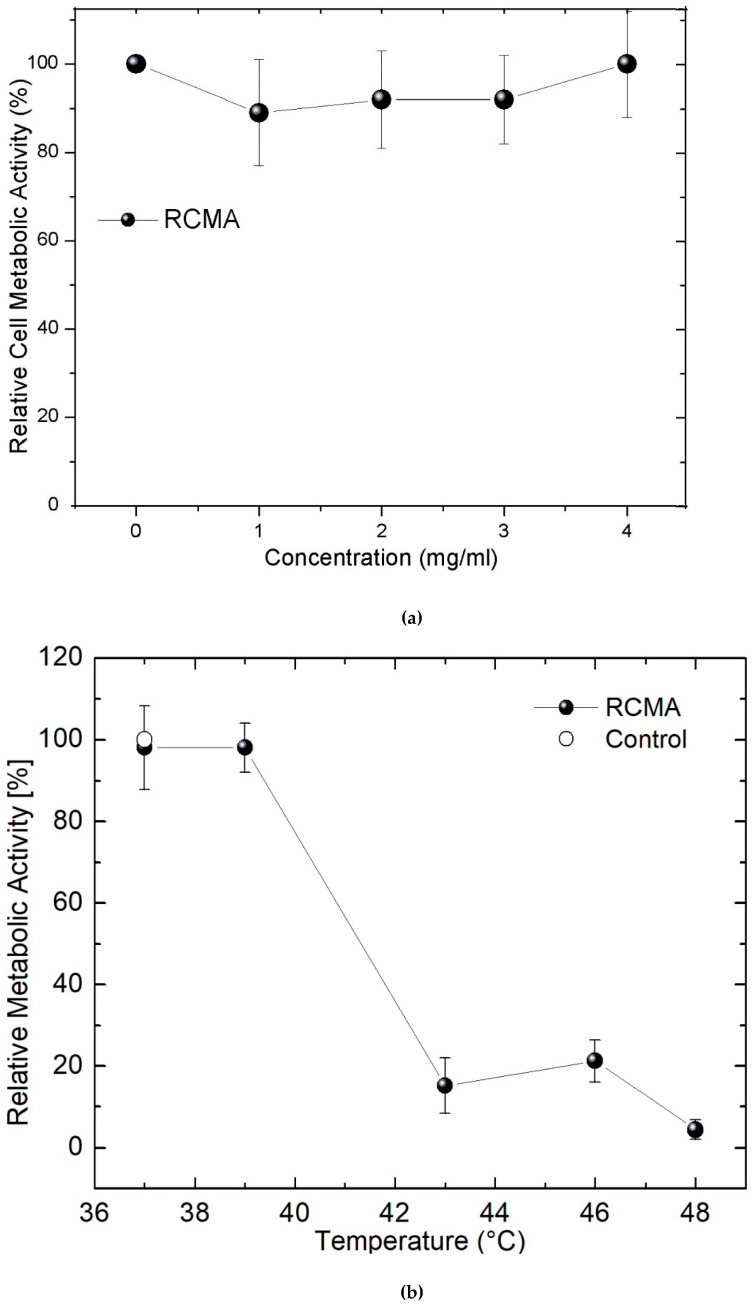
Relative metabolic activity of HT-29 cells (**a**) at different concentrations of coated MNPs and (**b**) after 20 min of magnetic field irradiation (using 3 mg/mL of MNPs, *f* = 530 kHz and *H* ranging from 12 kA/m to 20 kA/m).

The analysis of cytotoxicity of the ferrofluid is detailed in Figure 9a. It is important to highlight that the cells were exposed for 24 h to the coated MNPs which were precipitated on the monolayer of cells. This is the expected behavior because the pH of the culture medium is 7.4 and the corresponding Zeta potential is nearly −25 mV (see Figure 3b). In that plot, the correlation of the concentration of coated MNPs with the relative cell metabolic activity (RCMA) is estimated. This parameter is proportional to the increase of the optical absorbance of the formazan salts produced by the viable cells. Remarkably, no diminution was observed of the RCMA, even when employing concentrations up to 4 mg/mL. In general, the MNPs are endocytosed by the cells and can be modified by the lysosomal degradation mechanism. An excess of MNPs around the cells produces oxidative stress because the balance between ROS formation and the detoxification enzymatic system favors an increase in ROS levels. In this case, the CMNP does not produce dramatic disturbances to the cellular activity. The measurements of RCMA yield an interesting result because the *P* observed in Figure 5b,c can be modulated in magnetic hyperthermia trials by increasing the concentration of coated MNPs to reach adequate heating. According to the measurements of Figure 5a where (1 mg/mL of CMNP was used), a temperature increase higher than 8 °C can be expected (after 5 min) by increasing the concentration up to 3 mg/mL and applying *f* = 530 kHz with *H* ≈ 20 kA/m. Then, to heat the cell cultures using these parameters of the magnetic field, an initial desired temperature is set during the first 3–5 min, and a total of 20 min of electromagnetic irradiation is completed. In all the trials, the intensity *H* is manually controlled. In Figure 9b, the dependence of the induced temperatures on the relative RCMA is shown, and dramatic changes are measured when they are submitted to MHT (adding only 3 mg/mL of MNPs). Through careful observation of that plot, no initial diminution of the RCMA was obtained when the cells are heated to 37 °C (*H* ≈ 12 kA/m). At 39 °C (*H* ≈ 15 kA/m), the RCMA is approximately 98 ± 5%, and from there it is diminished almost linearly down to 5 ± 3% at 48 °C (*H* ≈ 20 kA/m). Moreover, about 80% of the cells died at nearly 43 °C (*H* ≈ 17.5 kA/m).

**Figure 10:**
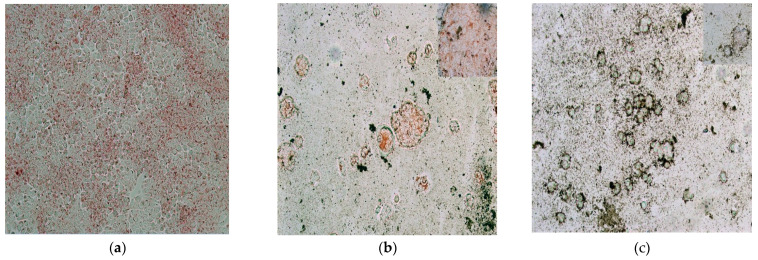
Red-stained HT-29 cells (mixed with 3 mg/mL of MNPs and after 24 h of incubation): (**a**) without MHT exposure; with MHT at (**b**) 43 °C, and (**c**) 48 °C.

The results obtained from NRU analysis are summarized in the images displayed in Figure 10a–c. Starting with the control cells, they exhibited 90% of confluence (Figure 10a) and are nearly uniformly red-stained, demonstrating the optimal function of the lysosomes. Similar behavior is observed when the cells with MHT are heated at 39 °C (data not shown). At 43 °C of MHT, the disruption effect of the cell membranes is observed, the confluence is drastically diminished to only 20% (Figure 10b), and, although the cells still appear to be red-stained, the partial union (island-like formations) among these can be highlighted. When the MHT induces 48 °C (Figure 10c), the cell cluster morphology is completely abnormal; the induced shapes of the cells are very different from those of the cells of the control sample. Indeed, the cell membranes are disintegrated, the fusion between adjacent cells is observed; the majority of the cells are detached, which reduces the confluence at approximately 5–10%, and finally, they no longer appear red-stained. Furthermore, another important observation from Figure 10b,c is the clear physical contact (such as adherence around the cells) between MNPs-cells and MNPs-cell debris. This could be an important starting point to determine the existence of apoptotic cellular death.

## 3. Materials and Methods

### 3.1. Synthesis of MNPs and Their Coating Procedure

The uncoated magnetic nanoparticles were prepared following the well-known coprecipitation method under an inert N_2_ atmosphere; this procedure is widely described in [17,21,24]. As a starting point, 30 mL 0.1 M of FeCl_3_ (Sigma-Aldrich, Burlington, MA, USA) and 15 mL 0.1 M Fe(SO_4_) × 7H_2_O (Fermont) were stirred at 350 rpm for 5 min, under a controlled temperature of 30 °C. Subsequently, 3.0 mL 5 M NH_4_OH (the precipitating agent) was slowly added at 2 mL/min. After 45 min of centrifugation (3000 rpm), a dark suspension of MNPs was formed within the liquid phase, which can be precipitated with the help of a large and strong magnet (15 kG on the surface). Then, the liquid was replaced by 300 mL of deionized water, and this procedure of magnetic precipitation and change of water was repeated four times, obtaining a ferrofluid of iron oxide MNPs suspended in water. To carry out the coating of the MNPs, a 40 mL aliquot of this ferrofluid was mixed with 20 mg catechol and sonicated for 70 min. Afterward, the resulting solution was shaken at 400 rpm for 24 h and the obtained suspension was centrifuged at 12,000 rpm. The pellet was resuspended in deionized water forming a stable colloidal aqueous suspension of catechol-coated MNPs (CMNP).

### 3.2. Physicochemical Characterization

The resulting colloidal solutions of uncoated MNPs and CMNP were poured into Petri dishes and dried in a vacuum oven (Thermo Scientific Lindberg/Blue M, Waltham, MA, USA) at 20 inHg vacuum, and 45 °C for 12 h. Subsequently, the FTIR of the resulting black powders were analyzed using a Thermo Scientific device in ATR modality (Nicolet iS5, Waltham, MA, USA). To obtain the FTIR spectrum of pure 1,2-Benzenediol, a sample of the reagent was taken without any previous treatment. In the same way, the dried samples with uncoated and coated MNPs were also analyzed via XRD using a Panalytical Empyrean device (Malvern, UK), sweeping its goniometer over the interval 5 ≤ 2θ ≤ 80° with 0.02° of fixed steps and ∆t = 30 s of sampling time.

TEM measurements were performed using a JEOL (JEM-2100, Tokyo, Japan) device, thus the samples of the ferrofluid CMNP were diluted at 1:100 and deposited onto FCF-200-Cu grids. Later, the samples were dried at room temperature and TEM micrographs are registered and stored.

A vibrating sample magnetometer VSM VersaLab (Quantum Design, San Diego, CA, USA) was used to determine the magnetization at room temperature, by sweeping a loop of magnetic field intensities *H* ranging from −30 kOe to 30 kOe. In addition, the ZFC-FC traces were registered by fixing *H* = 100 Oe covering the temperature interval from 50 K ≤ *T* ≤ 400 K. In these two types of magnetic trials, dried samples with 10 mg of coated and uncoated ferrofluids were analyzed.

A Zetasizer meter (ZS90, Malvern, UK) was employed to analyze the colloidal stability of the ferrofluid. Then, its capillary cells (DTS1070) were filled with aliquots of CMNP, which were mixed with some drops of 0.1 M NaOH to modify the pH during the interval 4.5 < pH < 10.

To determine the amount of catechol attached to the surface of the MNPs, thermogravimetric TGA measurements were registered by means of a TA analyzer (TGA-Q5000, New Castle, DE, USA). Therefore, an initial mass of dried CMNP and UMNP were deposited in a holder made of platinum, and later the samples were heated under an N_2_ atmosphere at 10 °C/min of heating rate covering the temperature range 50 °C < *T* < 500 °C.

A frequency variable induction heating system [37] (MX Pats. 65,340, Mx/a/2018/002848, Jalisco, Mexico) was employed to determine the response under alternating magnetic fields of the ferrofluid with CMNP. For these estimations, three samples of 1 mL were deposited into 2 mL Eppendorf tubes, which were placed inside the adiabatic region of the uniform magnetic field of the induction coil generator. The frequencies of magnetic field irradiation cover the 185 kHz < *f* < 530 kHz interval and the interval of amplitudes is 8.3 kA/m < *H* < 25 kA/m; simultaneously, a fluoroptic sensor (Luxtron-One, Santa Clara, CA, USA) records the temperature increase of the ferrofluid for 5 min.

### 3.3. Oxidative Capability Analysis of the Catechol-Coated MNPs Ferrofluid

ROS production was determined utilizing the procedure to degrade MB dissolved in water at room temperature [38]. Then, six samples were prepared in Eppendorf tubes partially filled with distilled water and their composition and label are summarized in Table 1. All the samples have a volume of 1 mL, the fraction used of CMNP and UMNP was 3 mg/mL, the volume of H_2_O_2_ was 250 μL (concentrated at 3.5%), and the concentration of MB was 5 μg/mL. Each sample was incubated at room temperature for 10 min to carry out the degradation of MB; immediately afterward, they were centrifuged at 10,000 rpm for 10 min, and the absorbance of the supernatants was registered using UV-vis spectroscopy at a wavelength of 665 nm (Mecasys Biopop, Daejeon, South Korea). Additionally, the absorbance of samples containing the supernatants with only UMNP and CMNP was determined and subtracted from the absorbance of the corresponding samples MB H_2_O_2_ UMNP, MB H_2_O_2_ CMNP, MB UMNP, and MB CMNP.

### 3.4. Cytotoxicity Analysis and Hyperthermia Assays of the Ferrofluid with CMNP

For the cell-biological experiments, the human cell line HT-29 (#HTB-38, ATCC, Manassas, VA, USA) was grown in DMEM (ATCC) supplemented with 10% of fetal bovine serum and 10 mM of 4-(2-hydroxyethyl)-1-piperazineethanesulfonic acid (HEPES buffer solution) at 37 °C, 4% CO_2_, and 95% of relative humidity. Approximately 1.5 × 10^5^ of cells were seeded onto a cover slide in 12-well-plates or single 2 mL-glass-well containing 1 mL of growth medium. For cytotoxicity analysis, after 24 h of incubation (reaching 90% of cell confluence), we added 1.0, 2.0, 3.0, and 4.0 mg/mL of CMNP in 12-well-plates; then, the cells were incubated for a further 24 h. Subsequently, the old medium was discarded and the cells were washed three times with phosphate-buffered saline (PBS); finally, 1 mL of fresh medium was added, and then the samples were ready for further analysis. For MHT experiments, after 24 h of incubation, 3 mg/mL of CMNP was added into the single glass-wells, similar concentrations of MNPs have been used by [39,40,41]. Then, the cells were incubated for a further 2 h to ensure close contact between the nanoparticles and the cells and to increase endocytosis prior to beginning the magnetic field irradiation. Then, the cells were heated for 20 min by applying an amplitude *H* with a fixed frequency. During the first 5 min of irradiation, each sample had reached the desired temperature (37, 39, 43, 46, and 48 °C), which was kept constant during the following 15 min. Immediately after irradiation, the cells were incubated for 24 h under standard conditions of temperature; following the incubation, the old medium was removed and the cells were washed up to five times with PBS before adding 1 mL of fresh medium. Afterward, the metabolic activity was quantified using the WST-test. Thus, 20 µL of WST-1 (Clontech, Mountain View, CA, USA) was added to each well with the cells; then, these were incubated for 3 h, and later, the medium was centrifuged for 1 min at 10,000 rpm (Eppendorf 5415 D, Hamburg, Germany) and the optical absorbance of the supernatants was measured at λ = 440 nm (Biopop, Mecasys, Korea).

For the neutral red uptake (NRU), 30 µL of 0.33% neutral red solution (Santa Cruz, CA, USA) was added to the wells. Following 4 h of incubation, the cells were washed with PBS to enhance the contrast; finally, as the cells were grown on cover slides, they could be easily observed under the microscope (Axioskop 40FL, Zeiss, Oberkochen, Germany). All tests were done in triplicate.

### 3.5. Qualitative Measurement of ROS

The rise of ROS production by cells was determined using 2,7-dichlorofluorescein diacetate (DCF-DA) at 20 µM, following the procedure modified by Siddiqui et al. [42]. This chemical compound is passively internalized into the cells within 20 min of incubation, where it is oxidized by the formed ROS, inducing a green fluorescence of the dichlorofluorescein (DCF). Initially, the adherent cells were seeded on cover slides in six-well plates and were then prepared and labeled, as summarized in Table 2. When the samples contained H_2_O_2_, the volume employed was 1 µL (3.5%). When the samples contain coated or uncoated MNPs, we used a concentration of 0.3 mg/mL. Before analyzing the cells, they were washed twice with PBS, then 10 µL of DCF-DA was added, and they were incubated for 20 min. The qualitative analysis of ROS was performed using the epifluorescence microscope (Axioskop 40FL) with a source of blue light and utilizing green light filters.

## 4. Conclusions

In this work, we have shown the suitability of carrying out magnetic hyperthermia experiments utilizing a ferrofluid of catechol-coated magnetic nanoparticles. This ferrofluid exhibits a relatively high power absorption density and very low cytotoxicity. The covering of the magnetic nanoparticles allows good colloidal stability and modifies their magnetic behavior, demonstrating a superparamagnetic scenario. Additionally, the calorimetric characterization employing induction heating can be modeled using the linear response theory with relatively good concordance.

Furthermore, the results of the Fenton reaction with MB and ROS analysis by fluorescein detection have demonstrated and confirmed that catechol-coated MNPs promote high ROS production. This means that this ferrofluid can induce an apoptotic effect on the cells caused by ROS while the magnetic hyperthermia treatment is carried out. Additionally, the high degradation of MB by the possible activation of H_2_O_2_ implies that our ferrofluid can be useful for removing contaminants from wastewater.

## Figures and Tables

**Table 1:** Composition of the six labeled samples prepared to degrade the MB.

Label	Content				
	H_2_O	MB	H_2_O_2_	UMNP	CMNP
MBB	√	√			
MB H_2_O_2_	√	√			
MB H_2_O_2_ UMNP	√	√	√	√	
MB H_2_O_2_ CMNP	√	√	√		√
MB UMNP	√	√		√	
MB CMNP	√	√			√

**Table 2:** Composition of the six labeled samples prepared to analyze the intracellular ROS.

Label	Content			
	DCF	H_2_O_2_	UMNP	CMNP
NC	√			
PC	√	√		
DCF H_2_O_2_ UMNP	√	√	√	
DCF H_2_O_2_ CMNP	√	√		√
DCF UMNP	√		√	
DCF CMNP	√			√

## Data Availability

Data of the compounds are not available from the authors.

## Sample Availability

Samples of the compounds are not available from the authors.
